# Atypical Presentation of Salzmann Nodular Corneal Degeneration as a Subepithelial Corneal Dystrophy: A Case Report

**DOI:** 10.7759/cureus.24328

**Published:** 2022-04-20

**Authors:** Premalatha Santhiran, Wan Haslina Wan Abdul Halim, Meng Hsien Yong

**Affiliations:** 1 Ophthalmology, Universiti Kebangsaan Malaysia Medical Centre, Kuala Lumpur, MYS

**Keywords:** subepithelial corneal dystrophy, degenerative disease, dry eye, superficial keratectomy, salzmann nodular degeneration

## Abstract

Salzmann nodular corneal degeneration (SNCD) is a rare, non-inflammatory, slowly progressive degenerative disease of the cornea. It is characterized by bluish-white nodules raised above the surface of the cornea. SNCD does not seem to consist of one clinical entity, which poses challenges to differentiate it from corneal dystrophies.

A 20-year-old Chinese female with a history of eczema and dry eyes presented with a complaint of itchiness in both eyes, watery eyes, and gradual blurring of vision in the right eye for two years. Upon examination, right eye vision was counting fingers, whereas the left eye’s best-corrected vision was 6/9. The anterior segment examination of the right eye showed generalized cornea haziness with superficial vascularization, while the left eye showed mild corneal haziness with no vascularization. Otherwise, both eyes had no signs of corneal infection, corneal scar, subepithelial nodular appearance, or allergic reactions, with no evidence of fluorescein staining. Anterior segment optical coherence tomography (AS-OCT) did not indicate subepithelial opacity. Subsequently, the patient underwent right eye superficial keratectomy under local anesthesia. Although clinically the patient was thought to have a form of subepithelial dystrophy, the histopathological report confirmed it to be SNCD. The best-corrected vision of the right eye improved to 6/12 post-keratectomy.

We report an unusual presentation of Salzmann’s corneal degeneration as subepithelial corneal haziness, which was treated successfully with superficial keratectomy.

## Introduction

Salzmann nodular corneal degeneration (SNCD) was first described in 1925 by Maximilian Salzmann as an eczematous keratoconjunctivitis [1]. The nomenclature subsequently changed from Salzmann’s nodular corneal dystrophy to Salzmann’s corneal degeneration [2]. It is a rare, non-inflammatory slowly progressive degenerative disease of the cornea, which presents as bluish-gray opacity varying in number and sizes [1,3]. It commonly involves the midperipheral to peripheral cornea anterior to Bowman’s layer [4,5]. Although the disease is usually idiopathic, it can be associated with an inflammation, trauma, and surgery. The diagnosis of SCND is solely clinical based, and the patient is usually asymptomatic [6]. It may be supported by high-frequency ultrasound biomicroscopy (UBM), in vivo confocal microscopy (IVCM), anterior segment optical coherence tomography (AS-OCT), or corneal topography and tomography. Histopathological analysis can show the definite diagnosis, as it typically shows thinning of epithelium overlying a nodule [6]. Proper diagnosis is important, as the disease generally has a good prognosis and functional outcomes with medical and surgical management [7]. In this report, we present a case of atypical SCND that clinically presented as subepithelial corneal dystrophy, but early intervention and histopathological aided us in arriving at the conclusive diagnosis.

## Case presentation

A 20-year-old Chinese female, a chronic soft contact lens user, with a history of eczema, dry eyes, limbal stem cell deficiency for the past six years, presented with a complaint of itchiness in both eyes, watery eyes, and gradual blurring of right eye vision for two years’ duration, which had worsened for the six months. The underlying ocular surface disease was well controlled with preservative-free artificial tears and topical cyclosporin. Otherwise, there was no significant history of ocular trauma or surgery.

**Figure 1:**
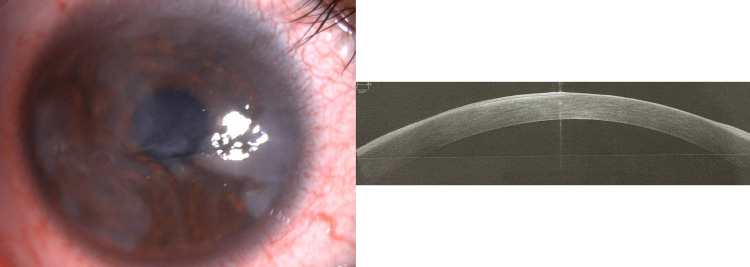
Pre-operative anterior segment photo and AS-OCT of the right eye AS-OCT, anterior segment optical coherence tomography

**Figure 2:**
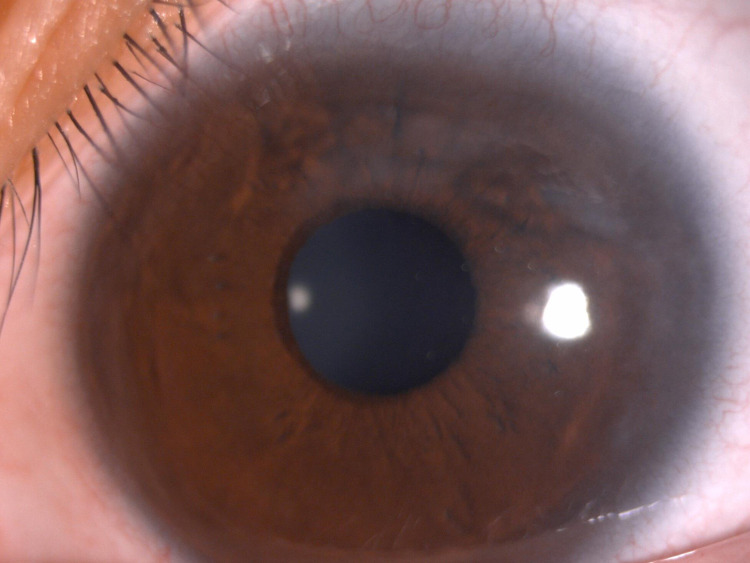
Photo of the anterior segment of the left eye

On examination, her right eye vision was counting fingers, whereas the left eye’s best-corrected vision was 6/9. Both eyelids showed no papillae. The anterior segment examination of the right eye showed generalized dense subepithelial cornea haziness with superficial vascularization (Figure 1). The left eye showed mild subepithelial corneal haziness (Figure 2).

**Figure 3:**
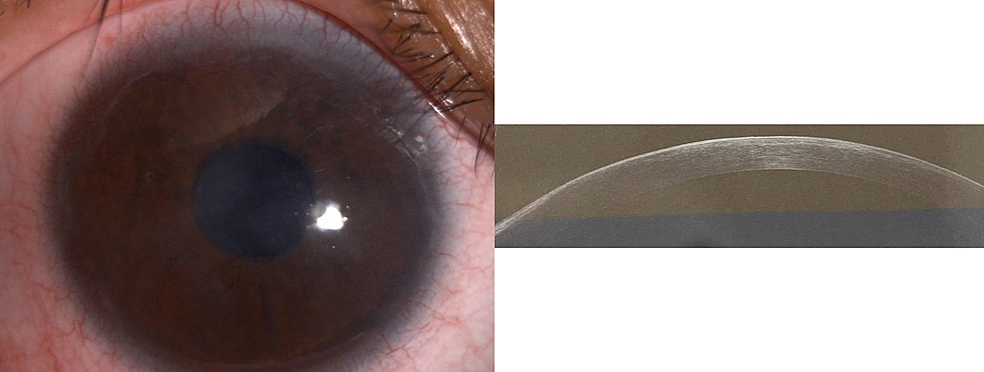
Post-operative anterior segment photo and AS-OCT of the right eye AS-OCT, anterior segment optical coherence tomography

There was minimal superficial punctate keratopathy on both cornea with no epithelial defect. There was no evidence of corneal infection, allergic reaction, ocular surgery, or trauma. Clinically, the patient was diagnosed as having bilateral subepithelial corneal dystrophy. AS-OCT showed generalized hyperreflective area confined to the subepithelial layer, with no overlying epithelial thinning nor nodular subepithelial thickening (Figure 1). Subsequently, the patient underwent right eye superficial keratectomy with subepithelial fibrosis removal under local anesthesia. Post-operatively, a bandage contact lens was applied until the epithelial defect healed, and the patient was started on topical steroids (prednisolone forte), and oral antibiotics (Cravit 0.5%) were started four hourly and then subsequently tapered off. The right eye’s best-corrected vision improved to 6/12 (Figure 3).

**Figure 4:**
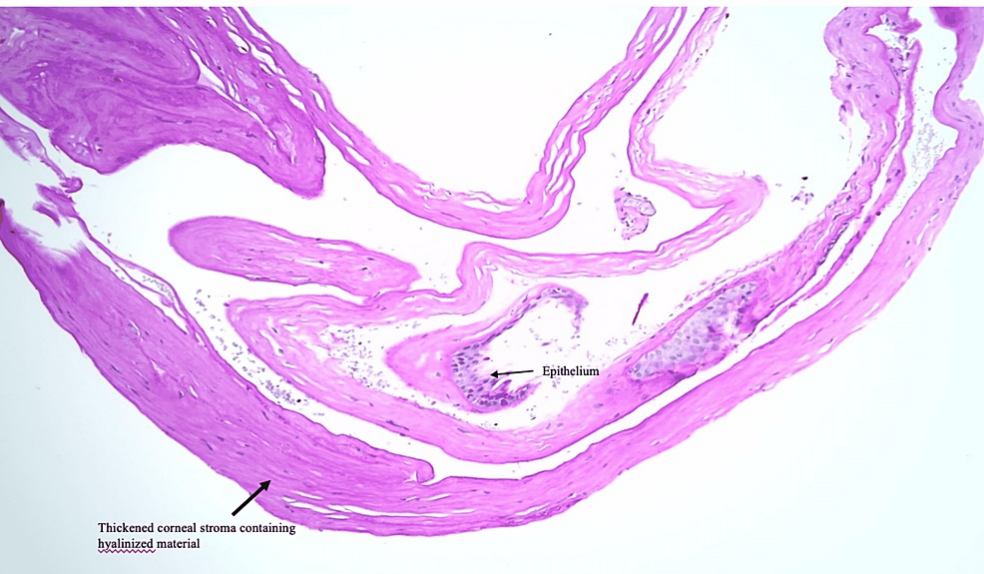
Histology slide of the debrided cornea of the patient with thickened corneal stroma, which contained deposition of hyalinized material.

Histologically, the corneal specimen was predominantly corneal stroma, which contained deposition of hyalinized material. The Masson trichrome and periodic acid-Schiff stains did not demonstrate the presence of intraepithelial material. No amyloid material was detected beneath the epithelium or within the stroma, which ruled out corneal dystrophy. These features instead are suggestive of Salzmann’s nodular corneal degeneration (Figure 4).

## Discussion

SNCD is a rare disease that commonly occurs in the fifth and sixth decades of life, although our patient presented much earlier in life. However, Hamada et al. did report that the disease can occur at any age range from 4 to 70 years. It is proposed that sex hormones may play a role in SNCD, thus predominantly affecting female patients. The androgen deficiency may lead to evaporative dry eyes and meibomian gland dysfunction, resulting in poor epithelial protection potentiating nodule formation as seen in our young female patient with dry eyes and ocular surface disease [3,8].

Multiple risk factors contribute to the occurrence of SCND, such as interstitial keratitis, vernal keratoconjunctivitis, meibomian gland dysfunction, dry eye, contact lens wear, ocular trauma, or previous ocular surgery [8]. Contact lens wear is associated with significant corneal epithelium thinning, lower levels of oxygen, and increased daytime corneal edema [9]. Yoon and Park have suggested that poor corneal epithelium protection can lead to SNCD formation [10]. Farjo et al. reported that 33.3% SNCD patients were contact lens wearers [7]. Risk factors identified in our patient included dry eye, limbal stem cell deficiency, and underlying ocular surface disease as primary risk factors. Besides that, she is also a chronic soft contact lens wearer, predisposing her to SNCD.

SNCD is usually asymptomatic or may present with foreign body sensation and reduced vision if the nodule or opacity involves the central visual axis [1]. Although the nodule may appear at any part of cornea, it is mostly seen at the midperipheral and peripheral parts [11]. Hamada et al. reported that in retrospective studies, 28% of the nodules were found in the superior-nasal quadrant, 23% in the superior-temporal quadrant, 20.5% in the inferior-nasal quadrant, 18% in the inferior-temporal quadrant, and 10% centrally located [8]. The nodules appear as bluish-gray subepithelial nodules, which vary in number and size. It may contain vessels or appear avascular [11]. Our patient presented with generalized subepithelial opacity with superficial vascularization, leading to a misdiagnosis of subepithelial dystrophy rather than SNCD, even though the patient had significant risk factors.

Imaging plays an important role in SCND as a monitoring tool. AS-OCT is a non-invasive tool that may help in the diagnosis of SCND. Nodules appear as hyperreflective subepithelial lesions overlying Bowman’s layer with thinned epithelium in AS-OCT [1,12]. Hurmeric et al. reported that AS-OCT usually demonstrates localized subepithelial triangular spikes highlighting the margins of the diffuse nodules with heterogeneous signal intensities in the central part of the nodules, whereas the posterior stroma and Descemet’s membrane are usually normal [13]. In our patient, AS-OCT was used as a primary imaging tool, which showed generalized hyperreflective subepithelial area with no nodules. The epithelium, Descemet’s membrane, and posterior stromal layer were normal. This feature also misled to the diagnosis of subepithelial corneal dystrophy rather than SCND. UBM and confocal microscopy can also play a role in the diagnosis with similar findings as AS-OCT [12], but none was performed in our patient.

Histologically, the findings of SNCD include the presence of epithelium and fibroblastic degeneration, absent or disrupted Bowman’s layer, hyaline plaques between corneal epithelium and Bowman’s layer, deposition of extracellular matrix, and irregular stroma collagen fibrils. The hyaline material does not stain for elastin, amyloid, or reticulin [4,10,14,15]. These findings were demonstrated in our case, which confirmed the diagnosis of SNCD even though it was initially thought to be subepithelial dystrophy based on clinical findings.

SCND can be managed conservatively or surgically. Preservative-free lubricants, warm compression, and lid hygiene may help in patients with dry eye. Topical steroids, topical non-steroidal anti-inflammatory drugs, and immunomodulators such as cyclosporin, may help improve ocular inflammation and ocular surface disease [6]. Graue-Hernández et al. stated that conservative management may prevent surgical intervention in 77% of cases [16]. If conservative management fails, surgical therapy is indicated in central visual axis lesion resulting in severe vision loss [8]. Surgical options include superficial keratectomy, lamellar keratoplasty, and penetrating keratoplasty. Additional adjuvant interventions such as mitomycin-C and amniotic membrane might be beneficial in SNCD [4,6,11]. Carroll et al. reported that 90% of cases showed visual improvement post-surgical intervention [17]. In our case, the patient was initially treated conservatively with preservative-free artificial tears and cyclosporin, as the patient had underlying dry eye and limbal stem cell deficiency. However, the lesion worsened with central involvement. Even though clinically she was diagnosed to have subepithelial dystrophy, she underwent superficial keratectomy because of severe vision loss and generalized subepithelial opacity involving the central visual axis. The debrided corneal tissue sent for histopathological examination confirmed SNCD. The vision improved dramatically from counting fingers to 6/12 post-surgical intervention. No adjuvant therapy was used in this patient because the lesion appeared to be superficial with no history of recurrence and the vision also improved post-operatively.

## Conclusions

SNCD has a good prognosis; thus, it is important to diagnose it early to achieve a good functional outcome with medical and surgical intervention. However, atypical presentation may sometimes lead to a delay in reaching the diagnosis. Currently, newer diagnostic imaging tools can also be utilized to play a role in aiding early diagnosis. Histopathological examination remains the gold standard to reach the conclusive diagnosis of SCND with atypical presentation and help differentiate it from corneal dystrophy.
